# Supplementary material for: Polygenic risk score validation using Korean genomes of 265 early-onset acute myocardial infarction patients and 636 healthy controls
Source: PLoS One. 2021 Feb 4;16(2):e0246538. doi: 10.1371/journal.pone.0246538 (PMC7861392; doi:10.1371/journal.pone.0246538)
Supplement: Attachment — Submitted filename: response_to_reviewers_PONE-D-20-27402.docx [file pone.0246538.s001.docx]

**Journal Requirements:**

1. Please ensure that your manuscript meets PLOS ONE's style requirements, including those for file naming. The PLOS ONE style templates can be found at

https://journals.plos.org/plosone/s/file?id=wjVg/PLOSOne_formatting_sample_main_body.pdf and

https://journals.plos.org/plosone/s/file?id=ba62/PLOSOne_formatting_sample_title_authors_affiliations.pdf

🡺 We have re-checked the journal’s style requirements and updated the manuscript as follows:

*We changed the file names of our manuscript and cover letter.

*In the author list part, we changed affiliation indication to number.

*In the author list part, we changed the symbol for equal contribution to the provided one (¶).

*In the affiliation part, we removed ZIP or postal codes.

*In the corresponding authorship part, we excluded physical addresses and left only email addresses.

*In the corresponding authorship part, we added the corresponding authors’ initials after the authors’ email addresses.

*In the manuscript, we removed the Keywords part from the title page.

*In the manuscript, we have changed the font size and headings to the PLOS ONE template.

*In the manuscript, we have changed the name of the Introduction section from Background to Introduction.

*In the manuscript, we have changed the name of the Material and methods section from Methods to Materials and methods.

*In the manuscript, we moved the Materials and methods section following after the Introduction section.

*In the manuscript, we have changed the citation of figures as Fig x.

*In the manuscript, we moved figure captions and table directly after the paragraph in which they are first cited.

*We removed the figure images from the manuscript and submitted as separate files.

*We removed the Declarations sections containing “Ethics approval and consent to participate”, “Availability of data and materials”, “Competing Interests”, “Funding”, and “Authors’ Contributions” from the manuscript.

*We converted the format of reference into Vancouver style.

2.We note that you have indicated that data from this study are **available upon request.** PLOS only allows data to be available upon request if there are legal or ethical restrictions on sharing data publicly. For information on unacceptable data access restrictions, please see http://journals.plos.org/plosone/s/data-availability#loc-unacceptable-data-access-restrictions.

In your revised cover letter, please address the following prompts:

a) If there are ethical or legal restrictions on sharing a de-identified data set, please explain them in detail (e.g., data contain potentially identifying or sensitive patient information) and who has imposed them (e.g., an ethics committee). Please also provide contact information for a data access committee, ethics committee, or other institutional body to which data requests may be sent.

b) If there are no restrictions, please upload the minimal anonymized data set necessary to replicate your study findings as either Supporting Information files or to a stable, public repository and provide us with the relevant URLs, DOIs, or accession numbers. Please see http://www.bmj.com/content/340/bmj.c181.long for guidelines on how to de-identify and prepare clinical data for publication. For a list of acceptable repositories, please see http://journals.plos.org/plosone/s/data-availability#loc-recommended-repositories.

We will update your Data Availability statement on your behalf to reflect the information you provide.

 🡺 The data presented here are available upon request since there is a legal restriction on sharing data publicly in Korea. We added detailed information for this issue in the revised cover letter as below:

“The data presented is under legal restriction since data contain potentially identifying or sensitive patient information. Therefore, raw sequencing data, individual genotype information, and clinical trait data will be available upon request and after approval from the Korean Genomics Center’s review board in UNIST. Information about the KGP and other data sharing can be found at [**http://koreangenome.org**](http://koreangenome.org)**/Cardiomics**.”

3. Your ethics statement should only appear in the Methods section of your manuscript. If your ethics statement is written in any section besides the Methods, please delete it from any other section.

🡺 We have deleted the Ethics Statement section in the Declaration section.

4.Thank you for stating the following in the Competing Interests:

[I have read the journal's policy and the authors of this manuscript have the following competing interests: C.K. and B.K. is an employee, and B.C.K. and J.B. are the CEOs of Clinomics Inc. B.C.K. and J.B. have an equity interest in the company. All other authors have no conflicts of interest to declare.].

We note that one or more of the authors have **an affiliation to the commercial funders** of this research study : Clinomics Inc

1. Please provide an amended Funding Statement declaring this commercial affiliation, as well as a statement regarding the Role of Funders in your study. If the funding organization did not play a role in the study design, data collection and analysis, decision to publish, or preparation of the manuscript and only provided financial support in the form of authors' salaries and/or research materials, please review your statements relating to the author contributions, and ensure you have specifically and accurately indicated the role(s) that these authors had in your study. You can update author roles in the Author Contributions section of the online submission form.

Please also include the following statement within your amended Funding Statement.

“The funder provided support in the form of salaries for authors [insert relevant initials], but did not have any additional role in the study design, data collection and analysis, decision to publish, or preparation of the manuscript. The specific roles of these authors are articulated in the ‘author contributions’ section.”

If your commercial affiliation did play a role in your study, please state and explain this role within your updated Funding Statement.

 🡺 We have re-checked Authors’ Contribution and added the following sentences “**The funder provided support in the form of salaries for authors C.K., B.K., B.C.K., and J.B., but did not have any additional role in the study design, data collection and analysis, decision to publish, or preparation of the manuscript. The specific roles of these authors are articulated in the ‘author contributions’ section.”** at the end of the Funding Statement as below:

“This work was supported by the U-K BRAND Research Fund (1.190007.01) of UNIST; Research Project Funded by Ulsan City Research Fund (1.190033.01) of UNIST; Research Project Funded by Ulsan City Research Fund (1.200047.01) of UNIST; Research Project Funded by Ulsan City Research Fund (2.180016.01) of UNIST. This work was also supported by the Technology Innovation Program (20003641, Development and Dissemination on National Standard Reference Data) funded by the Ministry of Trade, Industry & Energy (MOTIE, Korea). This work was also supported by internal funding of Clinomics Inc. **The funder provided support in the form of salaries for authors C.K., B.K., B.C.K., and J.B., but did not have any additional role in the study design, data collection and analysis, decision to publish, or preparation of the manuscript. The specific roles of these authors are articulated in the ‘author contributions’ section.**”

2. Please also provide an updated Competing Interests Statement declaring this commercial affiliation along with any other relevant declarations relating to employment, consultancy, patents, products in development, or marketed products, etc.

Within your Competing Interests Statement, please confirm that this commercial affiliation does not alter your adherence to all PLOS ONE policies on sharing data and materials by including the following statement: "This does not alter our adherence to  PLOS ONE policies on sharing data and materials.” (as detailed online in our guide for authors [http://journals.plos.org/plosone/s/competing-interests](about:blank)). If this adherence statement is not accurate and there are restrictions on sharing of data and/or materials, please state these. Please note that we cannot proceed with consideration of your article until this information has been declared.

 🡺 We have re-checked the Competing Interest Statement and added the following sentences “**This does not alter our adherence to PLOS ONE policies on sharing data and materials.”** in the middle of the Competing Interest Statement as below:

“C.K. and B.K. are employees, and B.C.K. and J.B. are the co-CEOs of Clinomics Inc. B.C.K. and J.B. have an equity interest in the company. **This does not alter our adherence to PLOS ONE policies on sharing data and materials.** All other authors have no conflict of interest to declare.”

Please include both an updated Funding Statement and Competing Interests Statement in your cover letter. We will change the online submission form on your behalf.

🡺 We have attached both an updated Funding Statement and Competing Interest Statement in the revised cover letter.

Please know it is PLOS ONE policy for corresponding authors to declare, on behalf of all authors, all potential competing interests for the purposes of transparency. PLOS defines a competing interest as anything that interferes with, or could reasonably be perceived as interfering with, the full and objective presentation, peer review, editorial decision-making, or publication of research or non-research articles submitted to one of the journals. Competing interests can be financial or non-financial, professional, or personal. Competing interests can arise in relationship to an organization or another person. Please follow this link to our website for more details on competing interests: [http://journals.plos.org/plosone/s/competing-interests](about:blank)

**Reviewers' comments:

Reviewer's Responses to Questions**

**Comments to the Author**

1. Is the manuscript technically sound, and do the data support the conclusions?

The manuscript must describe a technically sound piece of scientific research with data that supports the conclusions. Experiments must have been conducted rigorously, with appropriate controls, replication, and sample sizes. The conclusions must be drawn appropriately based on the data presented.

Reviewer #1: Yes

Reviewer #2: Partly

🡺 We have modified manuscript to accommodate reviewers’ suggestions. (see below point-by-point responses)

2. Has the statistical analysis been performed appropriately and rigorously?

Reviewer #1: Yes

Reviewer #2: No

🡺 We have presented responses to accommodate reviewers’ comments. (see below point-by-point responses)

3. Have the authors made all data underlying the findings in their manuscript fully available?

The [PLOS Data policy](http://www.plosone.org/static/policies.action#sharing) requires authors to make all data underlying the findings described in their manuscript fully available without restriction, with rare exception (please refer to the Data Availability Statement in the manuscript PDF file). The data should be provided as part of the manuscript or its supporting information, or deposited to a public repository. For example, in addition to summary statistics, the data points behind means, medians and variance measures should be available. If there are restrictions on publicly sharing data—e.g. participant privacy or use of data from a third party—those must be specified.

Reviewer #1: Yes

Reviewer #2: No

🡺 For the data availability issue, we have added the detailed reason for the restricted data availability with the contact point for the data request in the revised cover letter below.

“The data presented is under legal restriction since data contain potentially identifying or sensitive patient information. Therefore, raw sequencing data, individual genotype information, and clinical trait data will be available upon request and after an approval from the Korean Genomics Center’s review board in UNIST. Information about the KGP, present dataset (Cardiomics), and other related data sharing can be found at [**http://koreangenome.org**](http://koreangenome.org)**/Cardiomics**.”

4. Is the manuscript presented in an intelligible fashion and written in standard English?

PLOS ONE does not copyedit accepted manuscripts, so the language in submitted articles must be clear, correct, and unambiguous. Any typographical or grammatical errors should be corrected at revision, so please note any specific errors here.

Reviewer #1: Yes

Reviewer #2: Yes

**5. Review Comments to the Author**

Please use the space provided to explain your answers to the questions above. You may also include additional comments for the author, including concerns about dual publication, research ethics, or publication ethics. (Please upload your review as an attachment if it exceeds 20,000 characters)

Reviewer #1: The authors of this study aimed to determine whether the polygenic risk score (PRS) based on the whole genomic sequencing of 265 early AMI patients would be helpful in classifying patients. They also tried to confirm whether PRS would help predict cardiovascular events after PCI procedure. In the previously published CAD-related PRS studies, PRS is known to be a significant factor in patient classification. However, as this study includes a small number of Asian patients, it is not yet known whether PRS will help classify Asian patients with AMI. This study seems to be of great value in that it tried to find out whether PRS helps classify AMI patients in Asians. However, there are some questions about the analysis, so they are pointed out below.

First, it is difficult to understand what odds ratio 1.83 is for (page 7, line 17-18). Describe more clearly what odds ratio is for the outcome.

🡺 Thank you for pointing out the vague description on odd ratio in the manuscript. We have described the patient as “early-onset AMI (acute myocardial infarction)” to make the endpoint of comparison clear for the readers (line 6, on page 9).

Second, why didn't the authors put the AUC for the PRS itself in Fig 1?

🡺 The reason we did not put AUC for our PRS itself in Fig 1 is because we wanted to present and emphasize the contribution of the PRS to the conventional risk factor rather than showing the performance of PRS itself as a single score parameter. Therefore, we chose the current Fig 1 and placed the description for the performance of PRS itself at the start of the section rather than adding the performance to the figure.

Third, in the analysis of Fig. 2, is there a reason that the age classification was 45? In the case of the control group, the age distribution is from 29 to 57 years old. Is there a reason to include only 45 to 50 years old in the old age group? If the age group is set as above, only 72 control groups are included in the old age group. But don’t these settings make it seem like there are differences in statistics?

🡺 We used age 45 to classify/divide the samples for abtraining a most balanced number of patients between two age groups (For 25 < age ≤ 45 years, **130** patients. For 45 < age ≤ 50 years, **134** patients). Apart from that, if we consider the significant negative correlation between age and PRS in the patient group (Spearman’s rho = -0.14, *P* = 0.025), and the lack of significant correlation between age and PRS in the control group (Spearman’s rho = 0.03, *P* = 0.463), the **age-dependent accuracy** of PRS was expected to show the comparable tendency even in different settings as also reported from the previous study (PMID: 32068818).

Reviewer #2: Manuscript PONE-D-20-27402 Thank you for giving us the opportunity to review the manuscript Title: “Polygenic risk score validation using Korean genomes of 265 early-onset acute myocardial infarction patients and 636 healthy controls” A manuscript in which the author described The benefit of polygenic risk score (PRS) for classifying patients with CAD and predicting further events, the PRS in early-onset AMI genomes showed improvement in the identification and genomic screening of Korean patients in early life for health risk prediction.. We have some points we would like to refer:
Comments:

-In diabetic patient the author did not identify type (I or II) and therapy (oral or insulin) as it strongly affects MACE in follow up.
-Repeat revascularization related to many technical factors either stent (DES drug type) stent size (diameter and length) etc..
-Repeat revascularization should be identified either ( target lesion , target vessel or non-target vessel ) revascularization.
-It is not clear the time of revascularization after the onset of symptoms .
-AMI should be subgroup analysis between STEMI and non- STEMI group).
-Death should be identified either cardiac death or all causes of death
-Other MACE components are beneficial in identifying the atherosclerosis progression specialy in young age group as stroke reinfarction or stent thrombosis

🡺 Thank you for the comments. The factors you listed are known to be associated with follow-up events and insufficient information for retrospectively collected patients limited the range of present analysis. Although the previous PRS based follow-up event prediction study reported a high risk for all-cause mortality from the high PRS group with similar consideration to the present manuscript [PMID: 30571185], extended studies accompanied with detailed clinical factors that you have listed may be required through large-scale randomized control trials for the application of PRS in the real field. Therefore, we have added the sentences addressing the importance of considering detailed clinical factors for the application of PRS in the prediction of the subsequent events to reflect your consideration to readers as below (line 23, page 14 to line 2, page 15).

“And such a PRS application for the follow-up and treatment possibly will become more effective and precise if the confounding effect of clinical factors such as detailed information of diseases, drugs taking, method of treatment, outcomes, durations through onset, treatment, discharge, and follow-up are considered together.”

6. PLOS authors have the option to publish the peer review history of their article ([what does this mean?](https://journals.plos.org/plosone/s/editorial-and-peer-review-process#loc-peer-review-history)). If published, this will include your full peer review and any attached files.


If you choose “no”, your identity will remain anonymous but your review may still be made public.


**Do you want your identity to be public for this peer review?** For information about this choice, including consent withdrawal, please see our [Privacy Policy](https://www.plos.org/privacy-policy).

Reviewer #1: No

Reviewer #2: No
