# Supplementary material for: Polygenic risk score validation using Korean genomes of 265 early-onset acute myocardial infarction patients and 636 healthy controls
Source: PLoS One. 2021 Feb 4;16(2):e0246538. doi: 10.1371/journal.pone.0246538 (PMC7861392; doi:10.1371/journal.pone.0246538)
Supplement: Attachment — Submitted filename: response_to_reviewers_PONE-D-20-27402R1.docx [file pone.0246538.s002.docx]

**Comments to the Author**

1. If the authors have adequately addressed your comments raised in a previous round of review and you feel that this manuscript is now acceptable for publication, you may indicate that here to bypass the “Comments to the Author” section, enter your conflict of interest statement in the “Confidential to Editor” section, and submit your "Accept" recommendation.

Reviewer #1: All comments have been addressed

Reviewer #2: All comments have been addressed

2. Is the manuscript technically sound, and do the data support the conclusions?

The manuscript must describe a technically sound piece of scientific research with data that supports the conclusions. Experiments must have been conducted rigorously, with appropriate controls, replication, and sample sizes. The conclusions must be drawn appropriately based on the data presented.

Reviewer #1: (No Response)

Reviewer #2: Partly

🡺 We have modified the manuscript defining death as all causes of death to accommodate the reviewer's suggestion.

3. Has the statistical analysis been performed appropriately and rigorously?

Reviewer #1: (No Response)

Reviewer #2: N/A

4. Have the authors made all data underlying the findings in their manuscript fully available?

The [PLOS Data policy](http://www.plosone.org/static/policies.action#sharing) requires authors to make all data underlying the findings described in their manuscript fully available without restriction, with rare exception (please refer to the Data Availability Statement in the manuscript PDF file). The data should be provided as part of the manuscript or its supporting information, or deposited to a public repository. For example, in addition to summary statistics, the data points behind means, medians and variance measures should be available. If there are restrictions on publicly sharing data—e.g. participant privacy or use of data from a third party—those must be specified.

Reviewer #1: (No Response)

Reviewer #2: No

🡺 For the data availability issue, we have listed the kind of data available upon request and updated the reason for the restricted data availability with the contact point for the data request in the revised cover letter as below.

“Sequence raw data, individual genotypes, and clinical trait information are under legal restriction in Korea since the data contain potentially identifying or sensitive personal information. Therefore, the above data will be available upon request and after an approval from the Korean Genomics Center’s review board in UNIST. Information about the KGP, present dataset (Cardiomics) and other related data sharing can be found at <http://koreangenome.org/Cardiomics>.”

5. Is the manuscript presented in an intelligible fashion and written in standard English?

PLOS ONE does not copyedit accepted manuscripts, so the language in submitted articles must be clear, correct, and unambiguous. Any typographical or grammatical errors should be corrected at revision, so please note any specific errors here.

Reviewer #1: (No Response)

Reviewer #2: Yes

6. Review Comments to the Author

Please use the space provided to explain your answers to the questions above. You may also include additional comments for the author, including concerns about dual publication, research ethics, or publication ethics. (Please upload your review as an attachment if it exceeds 20,000 characters)

Reviewer #1: (No Response)

Reviewer #2: Manuscript PONE-D-20-27402R1 Thank you for giving us the opportunity to review the manuscript Title: “Polygenic risk score validation using Korean genomes of 265 early-onset acute myocardial infarction patients and 636 healthy controls” We have some points we would like to refer:
- AMI should be subgroup analysis between STEMI and non- STEMI group).
- Death should be identified either cardiac death or all causes of death
- Other MACE components are beneficial in identifying the atherosclerosis progression specialy in young age group as stroke reinfarction or stent thrombosis

🡺 Thank you for providing us with suggestions. Your point on subgrouping AMI (STEMI and non-STEMI) and outcomes from the patients (MACE, stroke reinfarction, or stent thrombosis) is good which could be easily overlooked in general PRS cardiovascular genomics studies [PMID: 32068818, 30571185, and 30586733]. We have now modified the manuscript by adding a statement that specifies the importance of taking into account of subgroups of disease and kind of outcomes further as below (line 23, page 14 to line 2, page 15).

“And such a PRS application for the follow-up and treatment will possibly become more effective and precise if the confounding effect of clinical factors such as detailed information of diseases, subgroups of disease, drugs taking, method of treatment, kind of outcomes, durations through onset, treatment, discharge, and follow-up are considered together.”

Also, we modified the death denoted in the manuscript as all cause of death (line 1, page 7 in the method section; line 4 to 6, page 11 in the result section).

7. PLOS authors have the option to publish the peer review history of their article ([what does this mean?](https://journals.plos.org/plosone/s/editorial-and-peer-review-process#loc-peer-review-history)). If published, this will include your full peer review and any attached files.


If you choose “no”, your identity will remain anonymous but your review may still be made public.


**Do you want your identity to be public for this peer review?** For information about this choice, including consent withdrawal, please see our [Privacy Policy](https://www.plos.org/privacy-policy).

Reviewer #1: No

Reviewer #2: No

[NOTE: If reviewer comments were submitted as an attachment file, they will be attached to this email and accessible via the submission site. Please log into your account, locate the manuscript record, and check for the action link "View Attachments". If this link does not appear, there are no attachment files.]

While revising your submission, please upload your figure files to the Preflight Analysis and Conversion Engine (PACE) digital diagnostic tool, <https://pacev2.apexcovantage.com/>. PACE helps ensure that figures meet PLOS requirements. To use PACE, you must first register as a user. Registration is free. Then, login and navigate to the UPLOAD tab, where you will find detailed instructions on how to use the tool. If you encounter any issues or have any questions when using PACE, please email PLOS at [figures@plos.org](mailto:figures@plos.org). Please note that Supporting Information files do not need this step.
